# Sparse Representation Based Multi-Instance Learning for Breast Ultrasound Image Classification

**DOI:** 10.1155/2017/7894705

**Published:** 2017-05-25

**Authors:** Lu Bing, Wei Wang

**Affiliations:** ^1^School of Information and Computer Science, Shanghai Business School, Shanghai 201400, China; ^2^Department of Science and Technology, Shanghai Municipal Public Security Bureau, Shanghai 200042, China

## Abstract

We propose a novel method based on sparse representation for breast ultrasound image classification under the framework of multi-instance learning (MIL). After image enhancement and segmentation, concentric circle is used to extract the global and local features for improving the accuracy in diagnosis and prediction. The classification problem of ultrasound image is converted to sparse representation based MIL problem. Each instance of a bag is represented as a sparse linear combination of all basis vectors in the dictionary, and then the bag is represented by one feature vector which is obtained via sparse representations of all instances within the bag. The sparse and MIL problem is further converted to a conventional learning problem that is solved by relevance vector machine (RVM). Results of single classifiers are combined to be used for classification. Experimental results on the breast cancer datasets demonstrate the superiority of the proposed method in terms of classification accuracy as compared with state-of-the-art MIL methods.

## 1. Introduction

Breast cancer is currently one of the highest incidence diseases in women and it has serious implications for the health of women. Medical ultrasound imaging plays an important role in diagnosis and treatment of breast cancer, and it has some characteristics, such as being noninvasive, economic, effective, safe, and convenient. It has become the preferred method for early detection of superficial organ diseases [1, 2]. However, ultrasonography is operator-dependent, and reading breast ultrasound (BUS) images requires well-trained radiologists. Even experts may have observer variation. In recent years, computer-aided diagnosis (CAD) technology has become a hot issue in the medical domain [3]. CAD can provide objective, quantification, and decision-making information about superficial organs such as tumors. It is helpful to eliminate misdiagnosis in clinical practice due to subjective factors. At present, there are two problems in the CAD system for ultrasound images [3, 4]. One is the fact that it is quite difficult to position tumors precisely due to the image quality, the error of which can affect feature extraction and classification performance. The other is the lack of study for classification method under the inaccurate condition. A number of effective feature extraction and classifier designing methods have been proposed to try to solve the above problems [5–7].

For breast ultrasound CAD systems, the tumor region is located as a Region of Interest (ROI), and the features are extracted from the ROI [7, 8]. Finally, the tumor is classified as benign or malignant. There are some problems in BUS images such as attenuation, speckle, shadows, and signal dropout [7, 8]. These characteristics make computer-aided segmentation of BUS images very difficult and cause large difference between the autosegmented result and real ROI [7]. Such difference will directly affect the final classification accuracy because the features such as shape and margin are dependent mainly on correctly located ROIs. Fractal dimension [9], cooccurrence matrix [10], and wavelet coefficients [11] have been widely utilized to derive discriminant features. To raise the accuracy of segmentation, image enhancement preprocessing which includes neighbor tissue suppression, background correction, Gamma transforming, and Gaussian smoothing are used to deal with ROI. Then, after segmentation by wavelet coefficients and prior medical knowledge, a mass feature extraction method based on concentric circles is used [23], which divides the image with different scales of concentric circles and also gets the topic features of masses.

Traditional learning methods are not available when facing the stage of classifying. In learning period, we only know the label of ROI, while the labels of the subregions in ROI are unknown. For solving this problem, ROI region can be viewed as a bag and its subregions can be regarded as the instances of the bag. Then, the problem is turned into a multi-instance learning (MIL) problem [12]. MIL was introduced by Dietterich et al. [12], which is originally proposed to solve learning problems with incomplete information of the labels of the samples. Many MIL methods have been widely used for applications, such as drug activity prediction, stock market prediction, natural scene classification, and content-based image retrieval [13–16]. For traditional learning, each training example is represented by a fixed-length vector of the features with known label, while in MIL, each sample is regarded as a bag (with labels) and consists of instances (without labels), and the number of instances in each bag can be different [7]. The object of MIL is to learn a model to classify new bags [13, 14]. In BUS images, we cannot simply consider that the points (instances) in a benign mass (bag) are all negative; and if only one point (instance) is positive, then the mass (bag) is malignant. In order to solve this specific problem, in [17], the generalized multi-instance learning (GMIL) was adopted. It considered that a number of instances could determine the corresponding bag's label. In this paper, a method based on sparse representation [18, 19] is used to convert this specific MIL problem to a single-instance learning problem. In order to improve classification performance, every single classifier is constructed with dictionaries of different sizes, and then the classifying results of single classifiers are combined.

The paper presents a novel method for automatic classification of BUS images of benign and malignant breast tumors based on sparse representation under the framework of MIL. The remainder of the paper is organized as follows. Sections 2–4 detail the proposed method. Experimental results are reported in Section 5. Finally, we conclude this paper in Section 6.

## 2. Image Enhancement

To make the segmentation more accurate and reduce the impact of normal tissue to tumor segmentation, enhanced processing is needed. Four procedures, neighbor tissue suppression, background correction, Gamma transforming, and Gaussian smoothing, are used [21, 22].

Neighbor tissue suppression is to solve oversegmentation and undersegmentation problems caused by shading problem due to some objective factors. We calculate suspected mass of the extracted ROI area and estimate radius *r*. Retain ROI unchanged and suppress pixels located outside the ROI area. The suppression amplitude is proportional to the distance to the center of the ROI:

(1) $$\begin{array}{c} L\left(\;x,y\;\right)=R\left(\;x,y,\sigma ,r\;\right)*I\left(\;x,y\;\right), \end{array}$$

where *L* is the processed ROI image after neighboring tissue suppression, *x* and *y* represent the horizontal and vertical coordinates of the center in ROI, respectively, *R* is suppressing amplitude, calculated by formula (2), and *I* is the original image of ROI:

(2) $$\begin{array}{c} R\left(\;x,y,\sigma ,r\;\right)=\left\{\begin{array}{cc} e^{\left(r^{2}-\left(x^{2}+y^{2}\right)\right)/2\sigma^{2}}, & if\;\;x^{2}+y^{2}>r^{2}\\ 1, & else, \end{array}\right. \end{array}$$

where *σ* is a constant for adjusting *R* that directly affects the degree of ROI segmentation. Select an appropriate *σ* to avoid undersegmentation or oversegmentation. When *σ* is equal to the difference between the side length of ROI and radius *r*, we can obtain ideal segmentation result.

Ideally, a suspected mass lies in the center of ROI, with high gray value in center and low gray value around. We regard ROI as a curved space, with each pixel of ROI represented by (*x*, *y*, *G*(*x*, *y*)), where *x* and *y* represent the location of current pixel; *G*(*x*, *y*) represents the gray value of the current pixel. The ROI is shaped into hills but often shows uneven background when located in the chest wall, skin lines, or nearby dense glandular tissue, making the hill region of the surface in ROI image not obvious and hard to segment [21]. Therefore, background correction needs to be introduced. Firstly, the least square fitting method is used to obtain a fitting plane that is equal to the initial size of ROI, and then calculate the difference between initial ROI and the fitting plane, and make linear transformation for gray value of each pixel in image. This method can keep high-frequency information of suspected mass in ROI, achieving relatively flat background, which is beneficial for the segmentation of ROI.

After neighbor tissue suppression and background correction, the impact from the chest wall, skin lines, dense glandular tissue, and other backgrounds is reduced, but some tissue background still connects to the suspected mass region, concentrated in a certain gray range. In order to solve adhesions problem of background tissue to the suspected tumors, we use Gamma transforming of nonlinear gray transform, the basic idea of which is to use the power function as correction function, transforming the narrower portion of grayscale range to a wider range so as to improve the image contrast:

(3) $$\begin{array}{c} G=cX^{r}, \end{array}$$

where **X** is input gray value. *r* < 1 indicates wider range transform for low grayscale area; *r* > 1 indicates wider range transform for high grayscale area. Suspected tumors and surrounded background tissue regions both locate in the high grayscale region of ROI; therefore we can take *r* > 1 in order to separate the gray distribution between them. Gamma transform can enhance contrast degree so as to make suspected tumor and background tissue enjoy different gray ranges.

As a linear smoothing filter, Gaussian filter is based on Gaussian function to select weights, having an effective elimination of high-frequency noise and maintaining image details [22]. Equation (4) shows the process of Gaussian smoothing:

(4) $$\begin{array}{c} L\left(\;x,y\;\right)=G\left(\;x,y,\sigma \;\right)*I\left(\;x,y\;\right), \end{array}$$

where *L*(*x*, *y*) represents ROI image denoised by Gaussian smoothing and *σ* is standard deviation; *x*, *y* represent pixel position of input image, *I*(*x*, *y*) is the processed image by the above-mentioned neighboring tissue suppression, background correction, and Gamma transform, *∗* is the convolution operator, and *G*(*x*, *y*, *σ*) is the Gaussian template, shown as the following formula:

(5) $$\begin{array}{c} G\left(\;x,y,\sigma \;\right)=\frac{1}{2\pi \sigma^{2}}e^{-{\left(x+y\right)}^{2}/2\sigma^{2}}. \end{array}$$

## 3. Feature Extraction Based on Concentric Circle Method

**Figure 1:**
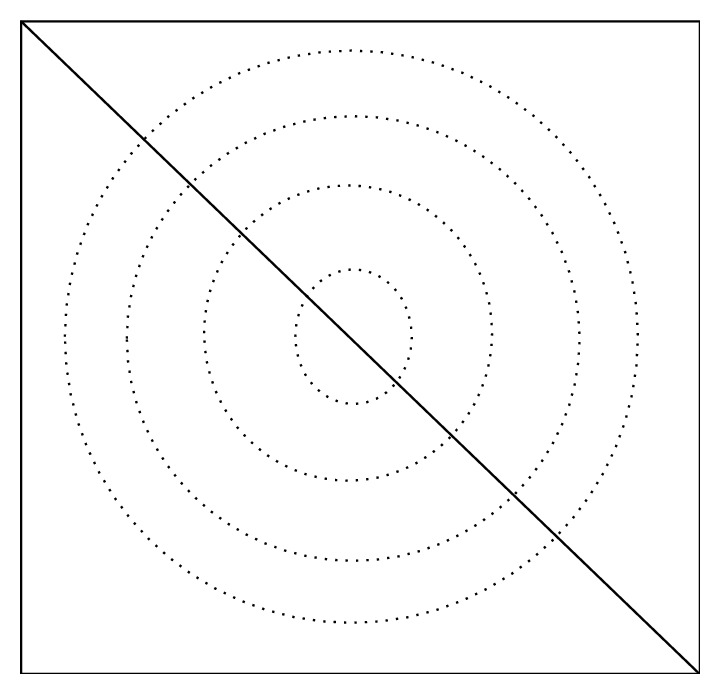
Concentric circle division.

For breast ultrasound CAD systems, the most remarkable features of a benign mass image are oval or round shape, circumscribed margins, and homogeneous internal echoes. The most remarkable features of a malignant mass image are speculated margins, irregular shape, ill-defined margins, and heterogeneous internal echoes [7, 23, 24]. The global features of tumors such as shape and margin can be extracted from segmented results. The local texture features are also effective in differentiating benign and malignant tumors. Spatial Pyramid Matching (SPM) model [25] is an algorithm performing image matching, identification, and classification based on Bag-of-Words (BoW) model and space pyramid [26]. In order to combine mammograms global features and local features to improve classification accuracy, feature extraction for breast masses based on concentric circle method is used. This method, which is based on SPM, can divide the image of similar characteristics into concentric circles, as is shown in Figure 1. Internal smooth mass can be divided into middle area, and the edge of tumors will be divided into the ring areas. Therefore, we can take full advantage of rounded features of breast tumors and separate feature points of the scale-invariant feature transform (SIFT) whose feature vectors are close to each other but belong to different areas.

In the experiment, firstly, size of mass image is adjusted so that the maximum length of the image is 1000 pixels; then the division of the subblock image is to divide the adjacent feature to the same area. Concentric circle dividing method is used to obtain the image diagonal line, dividing diagonal flat into *n* equal portions. Regard each length of* n* diagonal as radius and center of the image as center of circle to obtain middle circular region and outer annular region. We extract BoW characteristics of each block to obtain BoW features of *n* + 1 dimension:

(6) $$\begin{array}{c} F=\overset{n}{\underset{i=0}{\sum }}\lambda_{i}F_{i}, \end{array}$$

where *F*_0_ represents nonblocked BoW features, *F*_*i*_ represents BoW characteristics of the *i*th block, and *λ*_*i*_ indicates weight coefficient. We set the weight parameter as 1. Suppose that *M* is feature dimension of each image; the image has been divided into *N* concentric zones, and the final vector dimension can be obtained by *M* × (*N* + 1) dimensions. Suppose that BoW feature dimension of each image is 200; we use concentric circles of four layers for division, and the dimension vector is 1000.

In order to reduce dimension, this work refers to SPM-LDA method and combines latent Dirichlet allocation (LDA) [27] to obtain global and local features by full use of space information. LDA model was developed on the basis of pLSA (Probabilistic Latent Semantic Analysis), which can overcome the drawbacks such as the fact that the topic cannot be extended and the fact that parameters grow linearly with data size of pLSA. By using probability knowledge, we can extract latent topics linked between words. We select a topic by Dirichlet prior distribution. Since it has conjugate relationship with polynomial distribution, it can greatly reduce the computation and estimate parameters by a variety of methods. Probability density function of Dirichlet distribution is [26, 27]

(7) $$\begin{array}{c} Dir\left(\;\theta \;|\;\alpha \;\right)=\frac{\Gamma \left(\alpha_{0}\right)}{\Gamma \left(\alpha_{0}\right)\cdots \Gamma \left(\alpha_{k-1}\right)}\overset{K}{\underset{k=1}{\prod }}{\theta_{k}}^{\alpha_{k-1}}, \end{array}$$

where *θ* represents probability of latent topic in an image, *α* represents Dirichlet parameter, and Γ function is called Gamma function in the following form:

(8) $$\begin{array}{c} \Gamma \left(\;z\;\right)=\overset{\infty }{\underset{0}{\int }}\frac{t^{z}-1}{e^{t}}dt. \end{array}$$

We use LDA model combined with BoW model to extract the underlying topic features of the image, where an image can be seen as an article and visual words or SIFT feature points can be considered as the article word. Parameters control can ensure that images are organized according to a certain topic to give the image a certain topic distribution.

## 4. Sparse Representation Based Multi-Instance Learning

### 4.1. Multi-Instance Learning

Let *χ* represent the bag space and let *γ* be the set of class labels. In traditional supervised learning, the training data consist of samples {(*U*_1_, *v*_1_), (*U*_2_, *v*_2_),…, (*U*_*m*_, *v*_*m*_)}, where *U*_*i*_ ∈ *χ* is a bag and *v*_*i*_ ∈ *γ* is the label of *U*_*i*_. A model representing the function *f* : *χ* → *γ* is determined. On testing period, the object is to predict label *y* for new bag *U*. In MIL problem, the training data consist of bags and bag labels {(**B**_1_, *y*_1_), (**B**_2_, *y*_2_),…, (**B**_*m*_, *y*_*m*_)}, where **B**_*i*_ ∈ *χ* is a bag having a set of instances {*x*_1_^(*i*)^, *x*_2_^(*i*)^,…, *x*_*n*_*i*__^(*i*)^}, *x*_*j*_^(*i*)^ ∈ **B**_*i*_,   (*j* = 1,…, *n*_*i*_), *n*_*i*_ is the number of instances of the bag **B**_*i*_, and *y*_*i*_ ∈ {1,2,…, *C*}, with *C* denoting the number of classes [12, 13]. Every instance *x*_*j*_^(*i*)^ ∈ *ℝ*^*k*^ is a *k*-dimensional feature vector. Different bags contain different numbers of instances; hence, *n*_*i*_ may vary for different bags.

In our study, when a ROI is obtained and the features of subregions are extracted, the classification task can be converted into an MIL task. The subregions of ROI can be viewed as the instances and the ROI can be considered as a bag. The traditional MIL assumes that the positive bag has at least one positive instance and the negative bag has no positive instance. However, such thought is not available for classifying breast cancers. A malignant tumor is a group of cancer cells that may grow into surrounding tissues or spread to distant areas of the body. The tumor not only contains tumor cells but also contains other kind of tissues [7].

### 4.2. Sparse Representation

**Figure 2:**
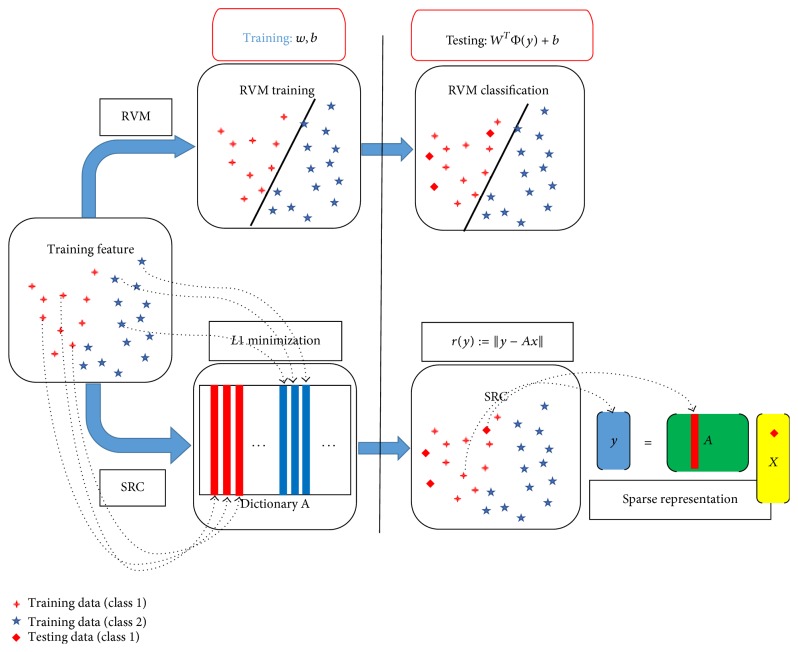
Comparison of the classification procedure and characteristics of the RVM and SRC.

Recently, sparse representation with a learned dictionary has been successfully applied to many practical problems [20, 28, 29]. In order to solve the above specific problem, a method based on sparse representation is used to convert this specific MIL problem to a single-instance learning problem that can be solved directly by single-instance learning methods. Each instance of a bag is represented by a sparse linear combination of all basis instances in the dictionary, and then the bag is represented by one feature vector which is obtained via sparse representations of all instances within the bag. After repeated dictionary learning, the sparse and MIL problem is converted to a single-instance learning problem that is solved by relevance vector machine (RVM) classifier. Comparison of the classification procedure and characteristics of the traditional RVM and sparse representation classification (SRC) is in Figure 2.

We denote data matrix *X* = {**x**^1^, **x**^2^,…, **x**^*m*^} ∈ *ℝ*^*k*×*m*^, where **x**^*i*^ represents instance and *m* = ∑_*i*=1_^*n*^*n*_*i*_. Denote **D** = [**d**^1^, **d**^2^,…, **d**^*s*^] ∈ *ℝ*^*k*×*s*^ as the dictionary matrix, where each column represents a basis vector of the dictionary and *s* is the dictionary size. Denote **α** = [**α**^1^, **α**^2^,…, **α**^*m*^] ∈ *ℝ*^*s*×*m*^ as the coefficient matrix, where each column is a sparse representation for a data sample. The target is to represent **x**^*e*^  (*e* = 1,2,…, *m*) as a sparse linear combination of vectors in the dictionary **D** [18, 29]:

(9) $$\begin{array}{c} \underset{D,\alpha }{\min}\overset{m}{\underset{e=1}{\sum }}\left(\;{\left\|\;x^{e}-D\alpha^{e}\;\right\|}_{2}^{2}+\gamma {\left\|\;\alpha^{e}\;\right\|}_{p}\;\right), \end{array}$$

where *p* = 1. *γ* > 0 is a regularization parameter. Usually, the higher the noise power is, the larger *γ* is. We solve the dictionary **D** via K-SVD dictionary learning method [20]. Once we get the dictionary **D**, we may solve the sparse representation **α**^*j*^ ∈ *ℝ*^*s*^ for each instance **x**^*j*^ of a bag **B** = {**x**^1^, **x**^2^,…, **x**^*J*^} [20].

We denote $\tilde{\alpha }=[\alpha^{1},\alpha^{2},\ldots ,\alpha^{J}]\in {\mathbb{R}}^{s\times J}$, where **α**^*j*^ is the sparse coefficient of instance **x**^*j*^ ∈ {**x**^1^, **x**^2^,…, **x**^*J*^}. We adopt max pooling function **b**_*h*_ = avr⁡{|**α**_*h*_^1^|, |**α**_*h*_^2^|,…, |**α**_*h*_^*J*^|} as bag features, where **b**_*h*_ is the *h*th element of **b** and **α**_*h*_^*j*^ is the matrix element at *h*th row and *j*th column of $\tilde{\alpha }$. Thus, every bag (ROI) is represented by an *s*-dimensional feature vector. Therefore, the MIL problem is converted into a single-instance problem.

In order to obtain higher classification accuracy and generalization performance, we combine multiple classifiers by different dimensional bags with different dimensional sparse representations based on different size of dictionaries [19, 30].

### 4.3. Algorithm

**Figure 3:**
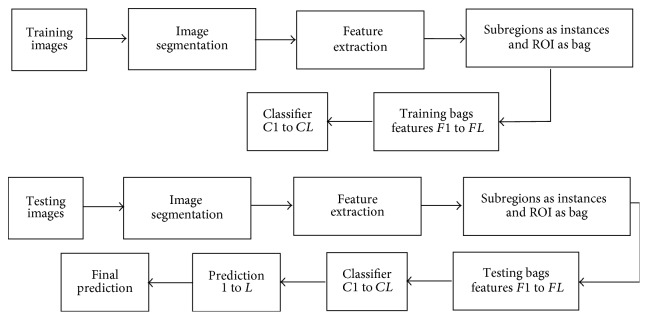
Flowchart of the proposed method.

The flowchart of the proposed approach is presented in Figure 3.

Finally, the procedure of our proposed method can be summarized in Procedure 1.

## 5. Experimental Results

In this paper, we use two widely used databases to verify our proposed method. One is the Wisconsin Breast Cancer Dataset (WBCD) [31] taken from the UCI Machine Learning Repository. It consists of 699 records. We gain a wholesome dataset with 239 malignant and 444 benign instances. Features are computed from a digitized image of a fine-needle aspirate (FNA) of a breast mass. The attribute information of WBCD is presented in Table 1. The other one is the Digital Database for Screening Mammography (DDSM) [32] by the mammographic image analysis research community. The database contains approximately 2500 studies. Each study includes two images of each breast, along with some associated patient information (age at time of study, ACR breast density rating, subtlety rating for abnormalities, and ACR keyword description of abnormalities) and image information.

### 5.1. Result and Analysis for Feature Extraction

**Figure 4:**
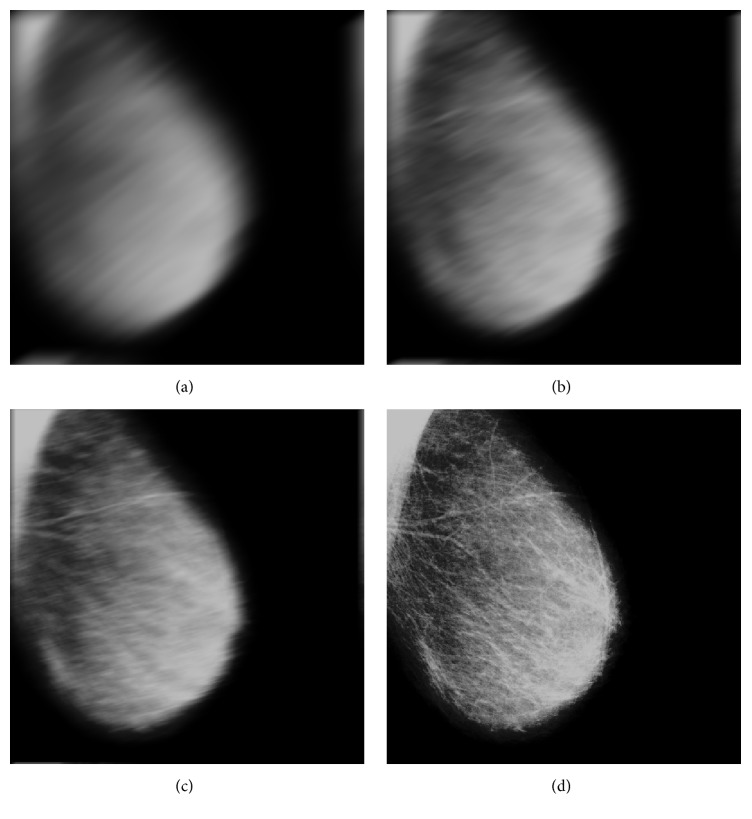
Image enhancement for ROI: (a) original, (b) after neighbor and background correction, (c) after Gamma transforming, and (d) after Gaussian smoothing.

The images presented here will be subsampled by 8 × 8 pixel size, that is, to improve the pixel size from the original 50 × 50 *μ*m up to 400 × 400 *μ*m.  Figure 4 shows experiment results of image enhancement for classical ROI. The image is left breast with the number code 3001 from DDSM database. In Figure 4, (b) is the image in (a) processed by neighbor and background correction, (c) is the image in (b) processed by Gamma transform, and (d) is the image in (c) processed by Gaussian smoothing.

**Figure 5:**
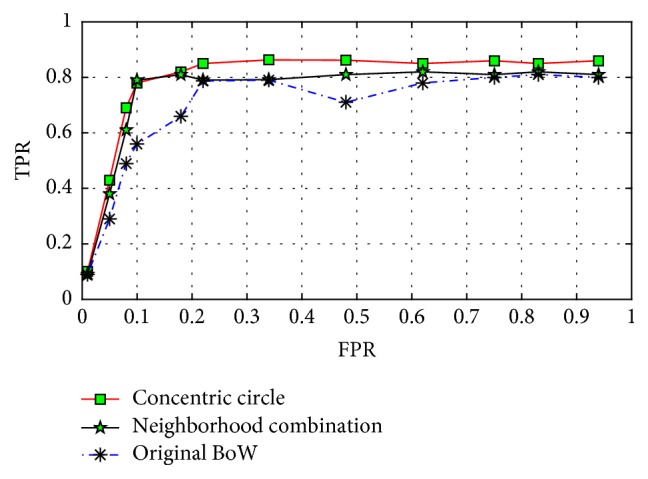
ROC curve comparison.

We study and compare experimental results of different concentric layered approaches. Results of the experiments are in Table 2. Divide the concentric circle into 4 layers, 8 layers, and 16 layers, respectively, and extract BoW features. The use of LDA will reduce the features to 80 dimensions. As can be seen, the basic classification accuracy rate is about 84.72%, and after four layers of concentric circles with the original BoW feature combinations, perform LDA dimensional reduction; we can obtain classification accuracy of 86.34%. This shows that the concentric method must contain the original BoW features to make BoW feature representation that contains not only global feature but also local feature; thereby LDA can fully extract topic feature, so that the final image features can obtain more robust representation. We also acquired receiver operator characteristic (ROC) curves shown in Figure 5. We can see that the concentric method has global and local features with the best performance.

### 5.2. Parameters Properties

In the experiments, three key parameters including regularization parameter *γ*, combining size *q*, and dictionary size set *S*{*s*1, *s*2, *sq*} are considered in order to evaluate the influence on the performance. Two subsets randomly selected from WDBC and DDSM are used for testing the convergence of *γ* and the accuracy on these two datasets is tested with different number of combining sizes.

**Figure 6:**
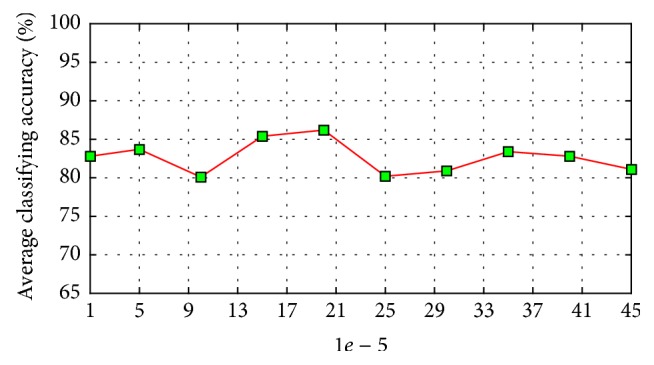
The recognition rates versus the parameter Gamma.

**Figure 7:**
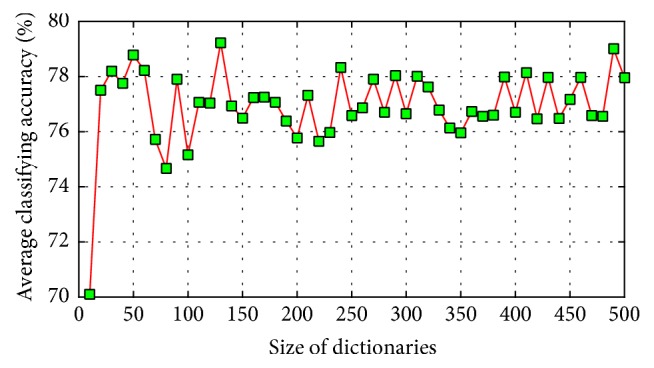
Classification accuracy of single classification trained from different size of dictionaries.

**Figure 8:**
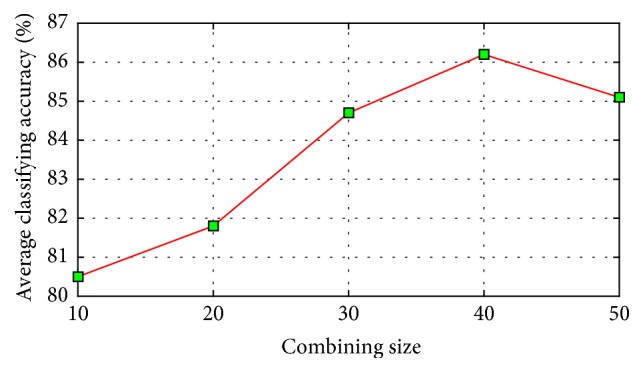
Classification accuracy of our method with different combining size.

The relationship between the parameter and the recognition rate of our method on WDBC dataset of a single run is shown in Figure 6, which shows that our method obtains good performance in a large range; well-performed recognition rates do not vary much with the change of the value of *γ*.  Figure 7 shows the average classification results of the single classifiers used by our method over ten randomly generated training sets and test sets; *s* varies from 10 to 500 with interval 10.  Figure 8 shows the average classification results of classifiers combining created by our method over ten randomly generated training and test sets, *q* varies from 10 to 50 with interval 10. In Figure 7, when the combining size is *q*, its corresponding dictionary size set is tuned from *S*{10, 20, …, 490, 500}. As can be seen from Figures 7 and 8, our proposed method outperforms the single classifiers in terms of classification accuracy. The single classifier obtains classification accuracies between 70.1% and 79.5%, while our proposed method obtains classification accuracies between 80.5% and 86.2%. These results demonstrate that the performance of the single classifier is relatively sensitive to the size of dictionary, while our proposed method is rather robust because of the considered combining thought.

### 5.3. Comparisons with Other MIL Methods

In our study, we compare the proposed method with other MIL algorithms, the Citation-KNN [13], expectation maximization methods with diverse density (EM-DD) [14] algorithms, and the method in [7]. Citation-KNN is an improved KNN algorithm suitable for MIL. EM-DD is an improved DD algorithm [16]. It estimates the label by using EM approach, which is still under the assumption of traditional MIL that there is at least one positive instance in a positive bag and there are all negative instances in a negative bag. But this assumption is not suitable for BUS image classification. The method in [7] is a novel MIL method for solving such task. First, a self-organizing map is used to map the instance space to the concept space, followed by the distribution of the instances of each bag in the concept space to construct the bag feature vector. Finally, a support vector machine (SVM) is employed for classifying the tumors. The experimental results have shown that the proposed method had much better performance, and it would be useful for CAD systems of BUS images.

The subject of this paper uses 900 mass images, in which benign and malignant image tumors are 500 and 400, respectively. The performance of the proposed classification strategy is evaluated by the classification accuracy. In order to verify the effectiveness of the method, in experimental analysis, 10-fold cross validation approach is used. The dataset is randomly divided into ten groups. Each time, one group is chosen for testing and the others are used for training. The experiments are independently performed 100 times and the average recognition rates on the test set are calculated and reported. The performance of the proposed feature extraction and classification strategy is evaluated by the classification accuracy. Define the number of correctly and incorrectly classified malignant tumors as true positive (TP) and false negative (FN) and the number of correctly and wrongly classified benign tumors as true negative (TN) and false positive (FP), respectively. The sensitivity (SE) is defined as follows: TP/(TP + FN). The specificity is defined as follows: TN/(TN + FP). The classification accuracy (ACC) is defined as follows: (TP + TN)/(TP + TN + FP + FN).

**Figure 9:**
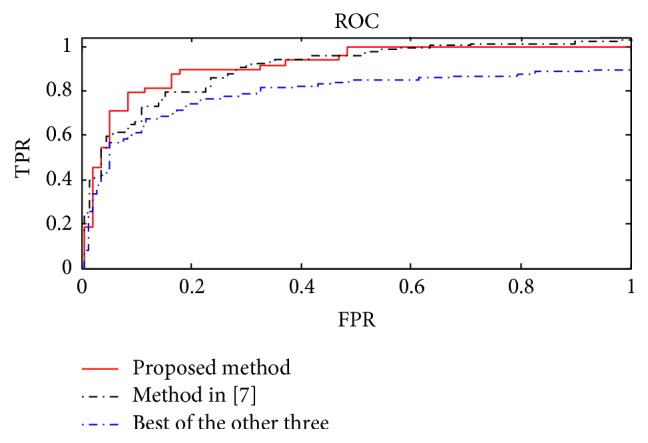
The ROC curves of the proposed method, the method in [7], and the best of three other methods, with the ROC area of 0.86267, 0.85973, and 0.79962, respectively.

In [7], the clustering methods including self-organizing map (SOM), *K*-means, and fuzzy *C*-means are used to transfer the instance space to the concept space construct, where SOM (49 neurons) and *k*-means (49 neurons) perform the best compared with other parameter settings. Therefore, we will use them in the experiment. Performance compared with four other methods is shown in Table 3. The ROC curves are also utilized to evaluate the performance of the proposed method as shown in Figure 9.

As can be seen in Figure 9, the AUC of the proposed method is higher than that of Citation-KNN, DD, and EM-DD and is slightly higher than that of the method in [7]. The method in [7] concentrates on local texture features, but our method considers global and local features. Also, the sparse representation based MIL contains natural discriminating information of instances and we combine multiple classifiers by different dimensional sparse representations so as to improve the classification problem, which is more robust. The above two aspects are regarded the reasons to obtain higher classification accuracy and generalization performance. However, it has to be pointed out that the time of dictionary learning and sparse representations' combination is a little longer than that of the classification method in [7].

## 6. Conclusions

In order to combine mammograms global features and local features and further improve classification accuracy, we use feature extraction for breast masses based on concentric circle method after image enhancement and segmentation. When a ROI is obtained and the features of subregions are extracted, the classification task can be converted into an MIL task. In order to adapt the MIL problem to single algorithms, a sparse representation based method has been used to compute bag features. The proposed method is utilized to classify tumors into benign and malignant ones. The experimental results show that the proposed method has better performance and may be useful for CAD systems of clinical BUS images. The limitations include the fact that parameters are obtained mostly by trial and error methods, and the time efficacy needs to be enhanced. Further, types of tumors should be more diversified so as to fully test the proposed method.

## Figures and Tables

**Procedure 1:**
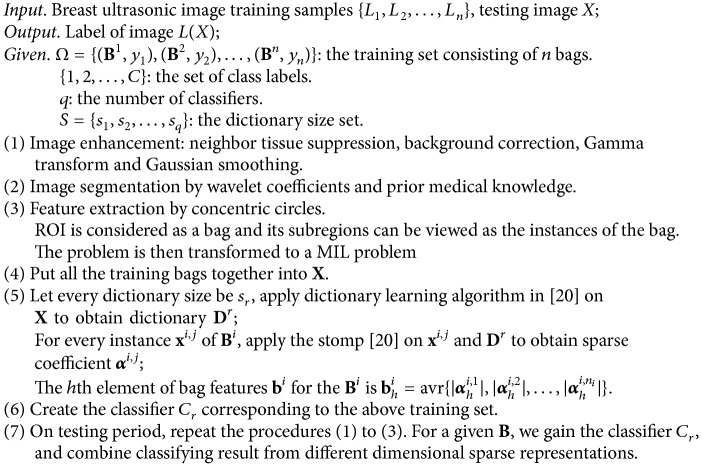
Sparse representation forbreast ultrasound image classification under the framework of MIL.

**Table 1:** Statistics of WBCD.

Attribute number	Attribute description	Minimum	Maximum	Mean	Standard deviation
1	Clump thickness	1	10	4.442	2.821
2	Uniformity of cell size	1	10	3.151	3.065
3	Uniformity of cell shape	1	10	3.215	2.989
4	Marginal adhesion	1	10	2.830	2.865
5	Single epithelial cell size	1	10	3.234	2.223
6	Bare nuclei	1	10	3.545	3.644
7	Bland chromatin	1	10	3.445	2.450
8	Normal nucleoli	1	10	2.870	3.053
9	Mitoses	1	10	1.603	1.733

**Table 2:** Classification accuracy (%).

Method	4 layers	8 layers	16 layers	BoW + 4 layers	2 layers
Feature dimension	80	80	80	80	400
Accuracy	84.16	84.45	84.72	**86.34**	86.11

**Table 3:** Performance compared with four other methods.

Method	TP	TN	SE (%)	SP (%)	ACC (%)
Citation-KNN	45	68	75.98	80.77	78.55
DD	50	64	80.26	78.18	75.02
EM-DD	47	72	76.44	81.25	79.85
Method in [7] with SOM (49 neurons)	51	59	78.34	81.22	84.95
Method in [7] with *k*-means (49 neurons)	51	62	77.27	80.65	84.56
Proposed method	53	68	79.21	79.91	86.25
